# Zinc transporters and insulin resistance: therapeutic implications for type 2 diabetes and metabolic disease

**DOI:** 10.1186/s12929-017-0394-0

**Published:** 2017-11-20

**Authors:** Shaghayegh Norouzi, John Adulcikas, Sukhwinder Singh Sohal, Stephen Myers

**Affiliations:** Faculty of Health, School of Health Sciences, University of Tasmania, Newnham Campus, Launceston, TAS 7250 Australia

**Keywords:** Zinc ions, Skeletal muscle, Cell signaling, Glycemic control

## Abstract

**Background:**

Zinc is a metal ion that is essential for growth and development, immunity, and metabolism, and therefore vital for life. Recent studies have highlighted zinc’s dynamic role as an insulin mimetic and a cellular second messenger that controls many processes associated with insulin signaling and other downstream pathways that are amendable to glycemic control.

**Main body:**

Mechanisms that contribute to the decompartmentalization of zinc and dysfunctional zinc transporter mechanisms, including zinc signaling are associated with metabolic disease, including type 2 diabetes. The actions of the proteins involved in the uptake, storage, compartmentalization and distribution of zinc in cells is under intense investigation. Of these, emerging research has highlighted a role for several zinc transporters in the initiation of zinc signaling events in cells that lead to metabolic processes associated with maintaining insulin sensitivity and thus glycemic homeostasis.

**Conclusion:**

This raises the possibility that zinc transporters could provide novel utility to be targeted experimentally and in a clinical setting to treat patients with insulin resistance and thus introduce a new class of drug target with utility for diabetes pharmacotherapy.

## Background

Insulin resistance (IR) is a common pathophysiological condition in which patients present with reduced insulin sensitivity and thus glucose intolerance, particularly in liver, adipose tissue and skeletal muscle [1]. This has significant implications for the patient, as they are unable to obtain to process the required energy from glucose to maintain cellular metabolic processes. IR is of major global concern as it is well-established as underpinning many chronic health conditions including type 2 diabetes mellitus (T2DM), obesity, cardiovascular disease polycystic ovary syndrome (PCOS), liver cirrhosis [2] atherosclerosis, hypertension, and stroke [3]. Moreover, given that IR usually precedes the development of T2DM and contributes to the progressive nature of this challenging and devastating disease, understanding the molecular mechanisms that lead to IR will help facilitate the development of novel therapeutic strategies to prevent or lessen disease progression. However, despite extensive ongoing research into IR, its molecular mechanism(s) of action remains largely elusive.

Recently, research on metabolic processes associated with IR and T2DM has revealed an exciting role for the biochemical and physiological role of zinc and the proteins that transport zinc in cells in diseases associated with abnormal cellular signaling [4]. Accordingly, zinc and the proteins that transport this metal ion have emerged as potential therapeutic targets for disease states associated with dysfunctional metabolism. For example, zinc in the diet and zinc transporter proteins that influence/regulate zinc metabolism are implicated in metabolic homeostasis in peripheral tissues (e.g. skeletal muscle and liver) that respond to insulin [4].

Zinc is ubiquitous in physiological systems, albeit, within tightly controlled parameters, and therefore suggests that atypical levels are likely to have significant biological and clinical effects on disease processes. Knowing how zinc transporter proteins and the storage of zinc in cells are involved in metabolic processes implicated in IR for example, may present opportunities to develop novel drugs targeting these transporters to prevent or treat IR and T2DM disease progression.

### Type 2 diabetes mellitus

Type 2 diabetes mellitus (T2DM) is devastating disorder characterised by hyperinsulinemia, hyperglycaemia, compromised energy metabolism and expenditure, and the progression of chronic illness and disease. T2DM is high complex involving both genetic predisposition and environmental factors. A major factor involved in a person’s susceptibility to T2DM can be linked through family history of diabetes. For example, Pacific Islander peoples are a unique population with especially high rates of T2DM [5]. The environment also plays a major role in the development of IR and T2DM with inactivity and poor nutritional status being two key factors [6].

#### Development of T2DM

The development of T2DM is preceded by IR, a disorder associated with hyperinsulinemia, glucose intolerance and dysfunctional energy metabolism [7]. A leading concern for people with IR is the progressive failure of pancreatic β-cell function (a major determinant of T2DM progression) and thus, compromised insulin secretion [8]. T2DM occurs primarily due to pancreatic β-cell failure, including disruption of β-cell function and mass [8]. Elevated blood glucose causes the pancreatic β-cells to produce more insulin resulting in hyperinsulinemia. Thus, pancreatic β-cells amplify insulin synthesis as well as insulin secretion pathways to overcome IR through an adaptive response called β-cell compensation [9]. Consequently, the failure of β-cells occurs in response to elevated insulin levels and thus elevated blood glucose which results in insulin insufficiency and overt diabetes. Accordingly, T2DM patients with loss of β-cell function will cease to live a normal life and will endure life-long pharmacological intervention, often with episodes of illness from unfavourable side-effects associated with the anti-diabetic treatments. Therefore, prevention strategies that take advantage of this “window of opportunity” (before β-cell failure) to prevent or lessen disease progression would have an enormous impact on the health and wellbeing of our communities.

#### Current drug treatments for T2DM

There are a range of medicines available to manage and treat IR and T2DM, however, side effects of these drugs have always been a foremost challenge in relation to the goal of pharmacotherapy [10] (Table 1). Yet, treatments for IR have not advanced significantly in the last few decades because of the inadequate knowledge about the pleiotropic effects that these drugs have on specific molecular targets [11]. Therefore, finding strategies to increase the efficacy and safety of therapeutic treatment for IR and T2DM is highly critical. In this context, research over the last decade has suggested a role for zinc in the treatment of T2DM [12]. For example, lowered zinc concentrations have been identified in some patients with T2DM [13] and zinc supplementation appears to improve the effectiveness of oral hypoglycaemic agents, decreasing blood glucose, triglycerides and inflammation in some patients [14]. However, many of these kinds of studies are not consistent in their findings and adds further complications in determining a role for zinc in these processes. Thus, it will be important to look at the role of zinc transporters and how they transport zinc within a cellular context during disease progression.

**Table 1:** Some common anti-diabetic therapies and their side effects

Current Therapies	Side Effects
Metformin (dimethylbiguanide)	Gastrointestinal intolerance and side effects [76, 77].
Sulphonylureas	Hypoglycaemia risk, weight gain [78], cardiovascular disease [79].
Incretin-based therapies	Arrhythmia [80], pancreatitis [81].
Thiazolidinediones	Risk of heart failure [82].
Dipeptidylpeptidase-4 inhibitors	Heart failure [83].
Sodium-coupled glucose co-transporter (SGLT-2) inhibitors	Dehydration and urinary infections in elderly patients [84].

### Zinc

Zinc is an essential trace element that is found in all parts of the body including the fluids and secretions, tissues and organs. Zinc is one of the most abundant trace metals (next to iron) in the human body, containing approximately 2–4 g [15]. The concentration of zinc in tissues is highest in the prostate (approximately 200 μg/ml), followed by the pancreas (approximately 40 μg/ml) and then muscle (approximately 50 μg/ml). In human plasma, there is approximately 14–16 μM of total zinc that is distributed to cells and subcellular organelles [15]. In multicellular organisms, almost all zinc is intracellular with the nucleus harbouring approximately 30–40%, the cytoplasm, organelles and specialised vesicles approximately 50%, and the cell membrane has about 10% [12]. Under normal cellular conditions there is no free zinc and therefore, the compartmentalization and distribution of cellular zinc is highly important and tightly controlled so that zinc homeostasis is maintained with an appropriate cellular concentration and physiological range. This is achieved by a family of zinc transporter proteins and metallothioneins [16, 17].

### Metallothioneins and zinc transporters

**Fig. 1:**
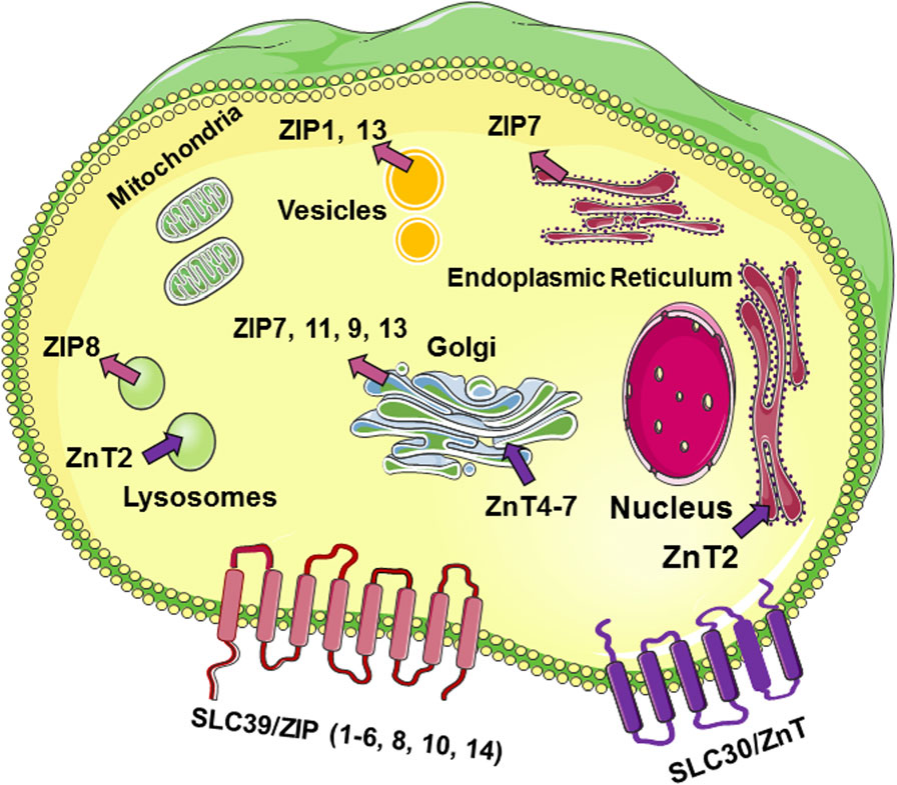
Cellular and subcellular localization of zinc transporters ZIP and ZnT. The primary localization of ZIP transporters (red arrow heads) and ZnT transporter (purple arrows) is shown representing the direction of zinc transport. Figure was produced using Smart Servier Medical Art, http://smart.servier.com/ and adapted from Kimura and Kambe (2016) [22]

Two families of zinc transporter proteins and zinc buffering proteins play a critical role in the influx, efflux, buffering and compartmentalization of zinc. These are the zinc transporters (ZnT proteins and Zrt/Irt-like ZIP proteins), and the intracellular zinc-binding metallothionein (MT) proteins [18]. MT proteins are a group of soluble low molecular weight metal binding proteins that buffer and translocate zinc within the cytosol [19]. The ZIP and ZnT^1^ zinc transporters belong to a family of transmembrane proteins that control zinc movement and thus zinc concentrations in cells. The ZnT family members (ZnT1–10) are involved in depleting cytosolic zinc by moving this metal ion into intracellular organelles or from the extracellular space while the ZIP family members increase cytosolic zinc by transporting this metal ion from outside the cell or from intracellular organelles (Fig. 1).

#### ZIP transporter family

The original member of the ZIP family of zinc transporters was identified in *Saccharomyces cerevisiae* (designated ZRT1) based on an amino acid sequence similarity to that of Irt1p, an Fe(II) regulated transporter from *Arabidopsis thaliana* [20, 21]. Consistent with the proposed role of ZRT1 in zinc uptake, zinc uptake was increased when this transporter was overexpressed in yeast cells. Similarly, a mutation that disrupted the function of ZRT1 led to reduced levels of zinc uptake and poor cellular growth in the mutant yeast strain [21].

**Fig. 2:**
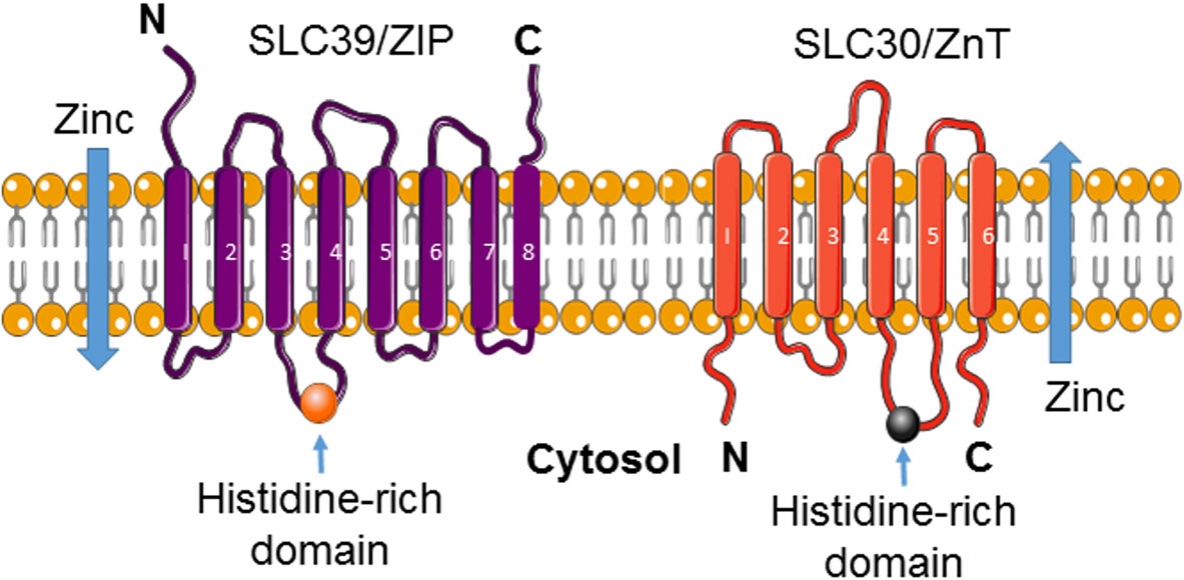
Predicted structure of the zinc transporters ZIP and ZnT. ZIP transporters are predicted to have eight transmembrane domains (TMDs) with a long histidine loop between TMDs 3 and 4. The ZnT transporters are predicted to have six TMDs with a histidine loop between TMDs 4 and 5. Figure was produced using Servier Medical Art, http://www.servier.com

The ZIP family of zinc transporters have a predicted eight transmembrane domain (TMD) structure and this is orientated with the N- and C-terminal facing the extracytosolic region. Many of these members have a predicted long histidine-rich loop region (HX_n_, *n* = 3–5) situated between TMD 3 and TMD 4 that is suggested to serve as a zinc-binding site (Fig. 2) [22, 23]. In mammals, there are at least fourteen ZIP transporters that have critical roles in the transport of zinc into cytoplasm from extracellular sources and intracellular storage compartments such as the Golgi apparatus and endoplasmic reticulum, when the cytosolic zinc is depleted. The ZIP transporters are regulated by intracellular and extracellular zinc concentrations, and hormones and cytokines. They are also expressed in several tissues and cell types and their proteins are localized to specific subcellular compartments and have been extensively reviewed elsewhere [24].

#### ZnT transporter family

The ZnT zinc transporters are a large family that include members with similar structural homology to bacteria, fungi, nematodes, insects, plants and mammals [25]. This family is predicted to have six transmembrane domains (TMDs) with a histidine-rich loop region between TMD 4 and TMD 5 (Fig. 2). The original mammalian ZnT was identified from a rodent cDNA library and was shown experimentally to confer resistance to zinc toxicity in baby hamster kidney cell lines [26]. Since the discovery of ZnT1, nine other members have been identified (designated SLC30A1–10). ZnTs are implicated in the transport of zinc into subcellular organelles and from the cytosol through the plasma membrane into the extracellular space [25]. Like the ZIPs, the ZnTs are regulated by intracellular and extracellular zinc, hormones, and cytokines and are extensively reviewed elsewhere [24].

### Zinc, zinc transporters and cellular signaling: A prelude to cell signaling and insulin resistance

Changes in zinc compartmentalization and availability in cells is typically detected and regulated by the intrinsic control of zinc transporter proteins. The cellular homeostasis of zinc is highly complex and there are several highly significant reviews on these processes [12, 15, 16, 27–30]. Accordingly, this section of the review aims to delineate the mechanisms by which zinc, and zinc transporters contribute to cell signaling and how these processes might provide insights into the molecular mechanisms implicated in disease processes such as insulin resistance and type 2 diabetes.

Zinc mimics the action of several molecules implicated in cellular metabolism including hormones, growth factors, and cytokines, and given the large number of mammalian zinc transporters that regulate zinc homeostasis, it is not surprising that this metal ion has been highlighted as a leading signaling molecule like calcium. Two modes of zinc signaling have been described. These are 1) early zinc signaling (EZS) and, 2) late zinc signaling (LZS). EZS, is a process that is independent of gene transcription and results in rapid changes in intracellular levels of zinc that occurs in minutes (the ‘zinc wave’ response) through the triggered release of zinc from subcellular organelles into the cytosol [31]. This phenomenon was first shown in studies in mast cells where an extracellular stimulation of these cells with the high affinity IgE resulted in a rapid increase in intracellular zinc from the endoplasmic reticulum within minutes [32]. LZS can also be triggered by extracellular stimuli but involves transcriptional-dependent changes in genes and thus proteins that are involved in zinc homeostasis such as storage proteins or transporters [33]. The importance of these two mechanisms of EZS and LZS highlight the diverse roles that this metal ion plays in processes that require rapid signals (such as metabolism) and more long-term functions such as cell differentiation and cell growth.

The defining role of zinc as a signaling molecule was shown in early studies in rat adipocytes where zinc could stimulate lipogenesis, independent, and additive to that of insulin [34]. Similarly, in rat adipocytes, zinc activated cAMP phosphodiesterase and the mobilization of glucose transporters to the plasma membrane, independent of insulin receptor kinase activity [35]. Since these studies implicating zinc as a signaling molecule, there is increasing evidence suggesting zinc acts in extracellular signal recognition [32], second messenger activity [36], protein kinase activity [37], protein phosphorylation [38], and transcription factor regulation [39]. These studies clearly highlight the role of zinc in signaling processes that are also associated with insulin-mediated metabolism.

The mechanisms of the insulin-mimetic action of zinc have been delineated in several studies however it is still unclear how these processes occur. One well-established mechanism of zinc action on cellular signaling events occurs through the inhibition of protein tyrosine phosphatase 1B (PTP1B). PTP1B functions as a negative regulator of insulin and leptin signaling transduction pathways [40]. Thus, the inhibition of PTP1B by zinc ions can prolong the insulin signal through the insulin receptor. Similarly, the ability of zinc to modulate glucose transport, glycogen synthesis, lipogenesis, to inhibit gluconeogenesis and lipolysis, and to regulate key elements of the insulin signaling pathway [41] suggests that this metal ion could provide therapeutic insight or utility in the management and/or treatment of insulin resistance. This is an interesting notion in a clinical context since patients that are insulin resistance have a “blunted” response to insulin and subsequent downstream cellular signaling responses. Therefore, the activation of cellular insulin signaling cascade that is critical to achieve glycemic control might involve zinc. However, the question remains as to whether zinc independently activates critical molecules involved in cellular signaling in the absence of insulin or whether zinc requires insulin for these processes.

#### Zinc transporters, cellular signaling and insulin resistance

Given the well-established role of zinc transporters in mediating the critical control of zinc homeostasis in cells, it will be important to further delineate their function in cell signaling events in the context of metabolic control. Currently, information is limited to what role the zinc transporters might play in cell signaling events in the context of insulin resistance. Therefore, extrapolation of studies from other cellular systems or disease states that have identified zinc transporters and thus zinc flux in facilitating cell signaling events might prove useful.

Studies accessing the role of zinc transporters in cellular signaling found that the zinc transporters ZnT5 and ZnT7 are responsible for loading zinc to alkaline phosphatases (ALPs) in the biosynthetic-secretory pathway in chicken B lymphocyte-derived cells [42]. These authors noted that mutant cells lacking both ZnTs resulted in a marked loss in ALP activity and this activity could be restored by overexpressing both ZnT5 and ZnT7. Similarly, the cooperative activity of ZnT1, ZnT4 and metallothioneins are required for the full activation of alkaline ALP in the early-secretory pathway [43]. Accordingly, the above studies demonstrate that ALP can be activated by the ZnT family of zinc transporters and therefore aid in the control of numerous cellular events.

In other studies, ZnT1 can regulate Raf-1 enzymatic activity in *Xenopus* oocytes and cultured mammalian cells [44]. Raf-1 plays a critical role in signal transduction in eukaryote cells where it phosphorylates and activates MEK1, a protein threonine and tyrosine kinase that activates the MAPK family members ERK1 and ERK2 [45]. ZnT1 was shown to bind to the amino end of the Raf-1 protein and promote kinase activation. Moreover, increasing the concentration of intracellular zinc inhibited Ras-mediated signaling through zinc blocking the ability of ZnT1 to bind Raf-1 [44] suggesting that Raf-1 activity requires functional ZnT1.

Of the ZIP transporters, ZIP14 is important in G-protein coupled receptor (GPCR)-mediated signaling through the maintenance of intracellular cAMP levels via suppression of phosphodiesterase activity [46]. In these studies, fasting gluconeogenesis is impaired in the livers of ZIP14 knockout mice which was attributable to changes in GPCR signaling processes. Similarly, in ZIP14 knock-out mouse chondrocytes, parathyroid hormone-related peptide (PTHrP)-mediated *c-fos* activity was significantly reduced. PTHrP stimulates the phosphorylation of cAMP response element-binding protein (CREB) which in turn induces the transcription of *c-fos* [47].

Studies in MCF breast cancer cell lines have identified that ZIP7 is essential in the redistribution of zinc from intracellular stores to the cytoplasm and subsequent zinc-induced inhibition of phosphatases [36]. Moreover, ZIP7 knock-down in these cells prevented the zinc-induced activation of epidermal growth factor receptor (EGFR), insulin-like growth factor-1 receptor (IGF-1R), and protein kinase B (AKT); key molecules implicated in cellular metabolism. In fact, ZIP7 has been coined the “gatekeeper” of cytosolic release from the endoplasmic reticulum (ER) and Golgi apparatus [48]. ZIP7 is phosphorylated by CK2 in MCF-7 cells and this activation leads to the “gated” release of zinc from the ER and the subsequent activation of multiple downstream signaling pathways including AKT and extracellular signal-regulated kinases 1and 2 (ERK1/2) [48].

ZIP7 is essential for the proliferation of intestinal epithelial cells and mice lacking this transporter in intestinal epithelium have massive apoptosis of transit-amplifying cells due to increased endoplasmic reticulum stress (ER) [49]. Similarly, studies identified that the phosphorylation of ZIP7 was increased in cardiomyocytes under hyperglycemia conditions and was implicated in driving ER stress [50]. Given that ZIP7 facilitates the release of zinc from the ER [48] and ablation of ZIP7 in mesenchymal stem cells led to the accumulation of zinc in the ER and subsequent ER dysfunction [51], it is plausible that ZIP7 could also be implicated in ER stress in type 2 diabetes. Undeniably, ER stress and the dysregulation of ER function in pancreatic beta cells are central in the pathogenesis of diabetes [52].

**Fig. 3:**
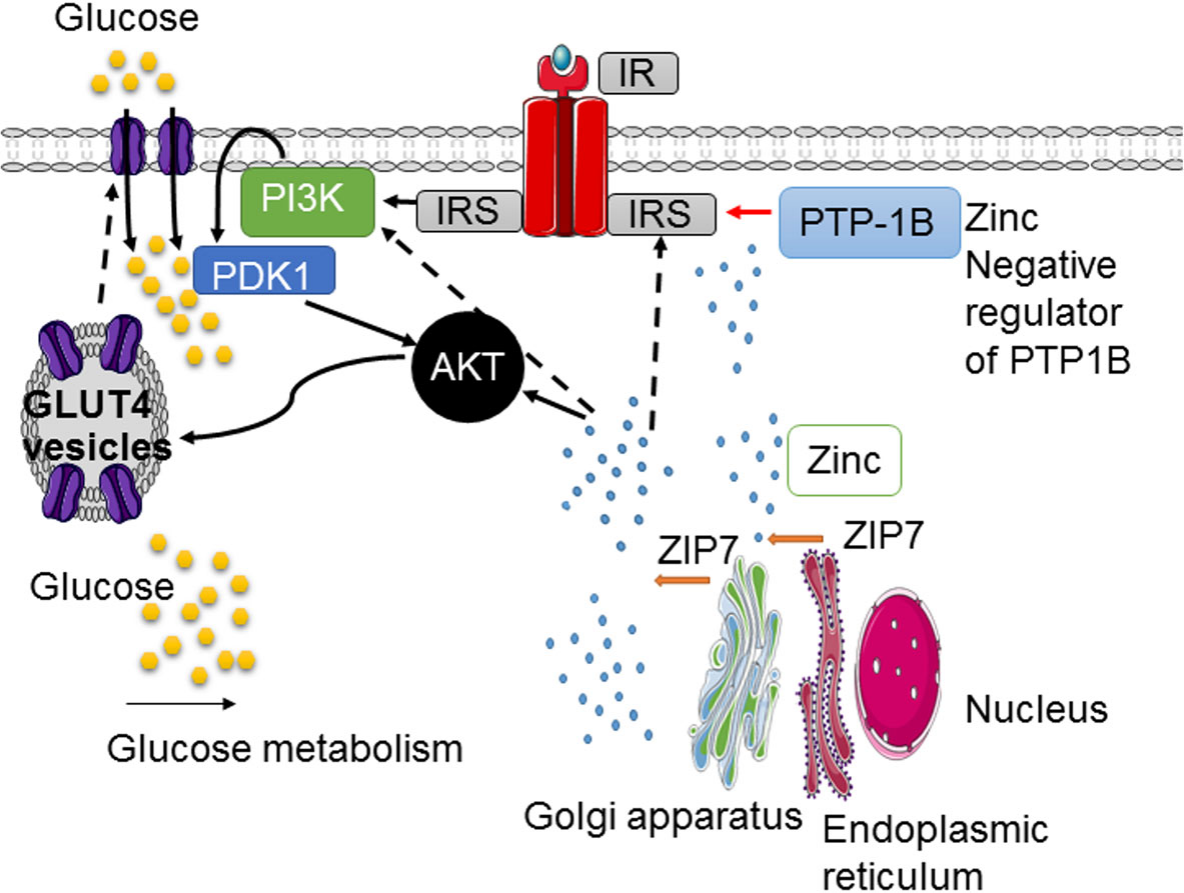
Potential role of ZIP7-mediated glucose metabolism in skeletal muscle. The ZIP7-gated release of zinc from the Golgi apparatus and/or the endoplasmic reticulum activates the phosphorylation of AKT and the subsequent mobilization of the glucose transporter GLUT4 which in turn brings glucose into the cytosol. Zinc also inactivates the negative regulation of insulin signalling, PTP-1B which allows the insulin signalling cascade process. The role of zinc activation of insulin receptor substrates (IRS) is not known (dashed lines)

Previously we have identified a role for ZIP7 in glycemic control in skeletal muscle [53]. Skeletal muscle acts as a major reservoir for zinc containing approximately 60% of total whole-body zinc [54]. Knock-down of ZIP7 in C2C12 mouse skeletal muscle cells led to a significant reduction in several genes and proteins involved in glucose metabolism including the insulin receptor (Ir), insulin receptor substrates 1 and 2 (Irs1 and Irs2), the phosphorylation of Akt, glucose transporter Glut4, and glycogen branching enzyme (Gbe) [53]. These data suggest that ZIP7 controls glucose metabolism via the phosphorylation of Akt and Glut4 mobilization (Fig. 3). It is not clear if reduced ZIP7 and thus reduced zinc levels leads to changes associated with the phosphorylation status of the insulin receptor substrates directly, or if reduced ZIP7 leads to inhibition of insulin receptor signaling via binding of PTP-1B. Studies have identified that the ZIPs play a major role in regulating cytosolic zinc homeostasis and insulin secretion [55]. In these studies, it was suggested that the zinc transporters ZIP6 and ZIP7 may have a role in insulin secretion in pancreatic beta cells via alterations in cytosolic and/or subcellular organelle-specific zinc pools. The down regulation of these transporters via knock-down studies led to a significant reduction in glucose-stimulated zinc uptake and oxidative stress in mouse islet cells [55]. These authors speculate that reduced expression of ZIP6 and ZIP7 may disrupt zinc homeostasis and thus produce defects in insulin secretion and beta cell viability that could potentially lead to the development of diabetes.

Recent studies have shown a relationship with ZIP13 and beige adipocyte biogenesis and thermogenesis [56]. In primary white preadipocytes isolated from white fat from ZIP13 null mice, there was a slight increase in common white fat genes but a significant increase in the gene expression of brown-fat specific genes and suggests that this transporter is implicated the inhibition of beige fat differentiation [56]. Moreover, these authors demonstrated that ubiquinated C/EBP-β was decreased in ZIP13 null mice. C/EBP is a major regulator of adipogenesis through the activation of genes essential for mitotic clonal expansion and thus, terminal adipocyte differentiation [57]. Accordingly, these studies suggest that ZIP13 deletion promotes beige adipocyte production and is associated with increased exergy expenditure, reduced diet-induced obesity and insulin resistance.

ZIP14 has been identified as a critical route for non-transferrin bound iron (NTBI) uptake into liver and pancreatic acinar cells and is essential for the development of liver iron overload in hemochromatosis [58]. Diabetes is frequently associated with hemochromatosis and patients with type 2 diabetes present with high ferritin levels which correlate with diabetic retinopathy [59]. Similarly, ZIP14 knock-out mice have enlarged pancreatic islets, low grade inflammation, and subsequent hyperinsulinemia and increased body fat which are characteristic of type 2 diabetes [60].

### Do the zinc transporters and zinc signaling play a role in insulin resistance and the progression of type 2 diabetes?

Zinc plays a major role in many aspects of cell signaling events in several physiological and pathophysiological processes. While it is well established that at least one zinc transporter (ZnT8) is critical for the compartmentalization, structure and secretion of insulin in beta cells of the pancreas [30], there is little information about the other family members in this context. Nonetheless, given the important role that zinc transporters play in delivering bioactive zinc to extracellular, cytosolic and subcellular milieu, and the action of this metal ion on cell signaling events, it is highly tempting to speculate that aberrant storage and release of zinc will result in unfavorable processes associated with insulin signaling and glycemic control.

#### ZnT8 and type 2 diabetes

Zinc is critical for the physiological role of insulin in the form of storage in the secretory granules of the pancreas as an inactive zinc-insulin hexamer [61, 62]. When the zinc-insulin hexamer is released into the blood circulation, a change in pH drives the dissociation of the complex into a bioactive monomer of insulin [63]. The zinc transporter that initializes zinc movement into insulin granules of the pancreatic β-cells is ZnT8. In fact, this transporter is almost exclusively localized in pancreatic β-cells and it is critical for the synthesis, storage and action of insulin [64].

Genome-wide association studies (GWAS) have discovered that a nonsynonymous single nucleotide polymorphism (SNP) in ZnT8 (rs13266634) encodes a C → T base substitution resulting in a change in the coded protein (p.Arg325Trp) and the production of two protein variants R and W of which the C allele (R variant) is associated with susceptibility to type 2 diabetes [65]. The frequency of the diabetes risk R allele is 91.5%, 71.7% and 56.7% in Africans, Europeans, and Asians, respectively [66]. Moreover, the corresponding at-risk R325 variant had reduced zinc transporter activity compared to the W325 ZnT8 in pancreatic beta-cell lines. Therefore, carriers of the R325 variant may have compromised packaging of insulin in pancreatic beta-cell granules [67]. In studies of 846 European individuals, each of whom had a parent with type 2 diabetes, it was demonstrated that homozygous carriers of the major C risk-allele variant had compromised pancreatic β-cell insulin secretion following an intravenous glucose load [68]. It was suggested by these authors that the function and/or production of ZnT8 in carriers of the C risk allele is reduced and therefore likely to contribute to compromised pancreatic beta-cell function.

Although GWAS studies have been successful in identifying variant ZnT8 alleles, it does not necessarily imply that the risk allele has a direct pathophysiological effect on beta-cell insulin secretion. A Meta-Analysis of Glucose and Insulin-related traits Consortium (MAGIC) was recently formed to conduct large-scale meta-analyses of genome-wide data for continuous diabetes-related traits in non-diabetics [69]. Meta-analyses were performed on approximately 2.5 M directly genotyped or imputed SNPs from twenty-one GWAS that were informative for fasting glucose (FG), fasting insulin (FI) and, pancreatic beta-cell function (HOMA-B) and insulin resistance (HOMA-IR) in non-diabetic participants [69]. It was identified that the risk allele for ZnT8 was associated with higher FG levels and an increase in two-hour glucose response in non-diabetics. Although these studies identified several genetic glycemic risk loci for type 2 diabetes, including ZnT8, not all loci are associated with pathological levels of glucose and type 2 diabetes risk.

More recent studies that sequenced approximately 150,000 individuals across five ancestry groups reported twelve rare protein-truncating mutations in ZnT8 which together explain a 65% reduced risk of developing type 2 diabetes [70]. It was found that a nonsense variant encoding (c.412C → T, p.Arg138*) heterozygosity yielded a 53% reduction in type 2 diabetes risk. Similarly, heterozygosity for the variant encoding p.Lys34Serfs*50 which is predicted to cause a frameshift and loss of all six ZnT8 transmembrane domains was associated with an 80% reduction in type 2 diabetes risk.

Recently, studies aimed to delineate the effect of dietary factor interactions with ZnT8 polymorphism (rs13266634) and the risk of developing metabolic syndrome found a significant interaction among omega-3 fatty acid consumption and ZnT8 in the context of metabolic syndrome, dyslipidemia, and abdominal obesity [71]. Participants with the CC genotype benefited more from the consumption of omega-3 fatty acids than carriers of the CT + TT genotypes. Carriers of the CC genotype had reduced risk of developing these disease states with increased consumption of omega-3 fatty acids. Moreover, the risk of abdominal obesity in the CT + TT genotype groups increased significantly with salty snack consumption but not in the CC homozygote carriers.

Studies to investigate the role of ZnT8 and glucose homeostasis has been established with ZnT8 null mouse models with global deletion of ZnT8 or pancreatic beta-cell specific ZnT8 deletion [72]. Most models resulted in impaired or unaltered glucose tolerance, however in ZnT8-depleted beta cells insulin granule abnormalities were observed, and this was concomitant with a loss of zinc release from secretory granules [73]. Similarly, ZnT8-specific deletion in beta cells resulted in reduced peripheral insulin concentrations despite an unexpected increase in insulin secretion from isolated ZnT8-depleted islets [74]. These authors suggest that secreted insulin from the pancreas in the ZnT8 knock-out mouse suppresses hepatic insulin clearance and dysregulation of this process could play a role in the pathogenesis of type 2 diabetes. A recent study [75] revealed ZnT8 deletion in mouse beta-cells resulted in a significant impairment in zinc release, normal or increased insulin secretion and subsequent impairment in glucose tolerance. Moreover, transgenic mice that overexpressed ZnT8 in beta cells showed a significant improvement in zinc release, lower levels of insulin secretion and improved glucose tolerance [75].

Given the pancreatic tissue-specificity of ZnT8 there is potential for this transporter to be amendable to therapy in the treatment of diabetes. However, targeting ZnT8 in the treatment of diabetes could prove to be highly complex. Although results from ZnT8 null mice suggest that increasing ZnT8 could improve insulin secretion and glycemic control, loss of function mutations in ZnT8 suggest a protective role for this transporter in type 2 diabetes. While ZnT8 plays a critical role in insulin physiology, and over the last decade or so this transporter has been given much attention for its role in diabetes, other zinc transporters have not had the same focused attention until recently. Apart from the many studies on zinc signaling in cells and the insulin-mimetic action of zinc, it is unclear which zinc transporters are involved in initiating these signaling processes. Clues from studies on the role of ZIP7 as the “gate-keeper” of zinc release from the Golgi apparatus [48] and subsequent ZIP7-mediated cell signaling events in skeletal muscle [49] no doubt place this transporter in an important position for further studies. Identifying how the zinc transporters are implicated in zinc signaling events that are amendable to insulin signaling processes in insulin resistance may help elucidate novel therapeutic options for the treatment of early diabetic symptoms and thus the long-term management of this disorder and associated type 2 diabetes.

## Conclusion

Zinc is an essential metal ion that is ubiquitous in many metabolic and physiological processes. The emerging role of zinc as an insulin mimetic in maintaining cellular function suggests that atypical levels, and aberrant compartmentalization, transport and storage of zinc will have biological effects that could be amendable to clinical intervention. Although current understandings on the role of zinc transporters in insulin resistance is not available, and this knowledge is only just emerging in type 2 diabetes, it is clear from studies on ZnT8 that this family of transporters has utility for the development of novel diabetic therapies. While ZnT8 plays a significant role in insulin biology and therefore represents an attractive target for diabetes therapy, the other members of the zinc transporter family in diabetes are less defined. However, we can speculate from the information presented in this review that the other transporters are involved in processes that facilitate insulin signaling and glycemic control and therefore could offer exciting new targets that are amendable to therapeutic intervention in the treatment of diseases associated with insulin resistance and type 2 diabetes.

## Footnotes

1 For brevity, the terms ZIP and ZnT will be used throughout.
